# Parasitic Fauna of *Lepus europaeus* and *Lepus timidus* in Kazakhstan: Parasitological Profile and Molecular Identification

**DOI:** 10.3390/biology14081083

**Published:** 2025-08-19

**Authors:** Vladimir Kiyan, Ainura Smagulova, Nurassyl Manapov, Karina Jazina, Rabiga Uakhit, Aitbay Bulashev, Lyudmila Lider, Sergey Leontyev

**Affiliations:** 1 Laboratory of Biodiversity and Genetic Resources, National Center for Biotechnology, Astana 010000, Kazakhstan; smagulova0114@gmail.com (A.S.); manapovbio@gmail.com (N.M.); dzhazinak01@mail.ru (K.J.); erken.uakhitrabiga@gmail.com (R.U.); leontyevs@yandex.kz (S.L.); 2 Scientific Center for Biological Research, Astana 010000, Kazakhstan; 3 Department of Veterinary Medicine, S. Seifullin Kazakh Agrotechnical Research University, Astana 010000, Kazakhstan; aytbay57@mail.ru (A.B.); con_80176@mail.ru (L.L.)

**Keywords:** *Lepus europaeus*, *Lepus timidus*, endoparasites, helminths, prevalence, phylogenetics, wildlife parasitology, Kazakhstan

## Abstract

Hares (*Lepus europaeus* and *Lepus timidus*) play important ecological and economic roles in Kazakhstan; however, data on their parasitic fauna are scarce. This study aimed to assess the diversity, prevalence, and genetic characteristics of endoparasites in wild hare populations from central and northern Kazakhstan. From November 2022 to February 2025, 107 hares (*L. europaeus*, n = 46; *L. timidus*, n = 61) were collected in the Akmola and Karaganda regions. Parasitological and coprological analyses were conducted. Genetic and phylogenetic analyses were performed using partial sequences of the mitochondrial *cox1* and *nad1* genes and a partial 28S rDNA region to assess the genetic diversity and evolutionary relationships of selected parasite genera. Seven parasite taxa were identified: *Mosgovoyia pectinata* (6.5% in *L. europaeus*, 11.5% in *L. timidus*), *Cysticercus pisiformis* (4.3% in *Le*, 3.3% in *Lt*), *Coenurus serialis* (3.3%, only in *Lt*), *Passalurus ambiguus* (6.5% in *Le*, 4.9% in *Lt*), *Nematodirus leporis* (36.6% in *Le*, 13.4% in *Lt*), *Eimeria* spp. (9.1% in *Le*, 3.8% in *Lt*), and *Trichuris leporis* (1.9%, only in *Lt*). Genetic methods confirmed species identity and revealed phylogenetic relationships. This is the first genetically confirmed survey of endoparasites in hares in Kazakhstan. The results contribute valuable data to regional parasitology and support the need for continued wildlife health monitoring.

## Simple Summary

Hares are important wild animals in Kazakhstan, both for the environment and for local hunting traditions. Like all animals, hares can carry parasites that may affect their health and the health of other animals and people. However, little was known about the species and spread of these parasites in hares living in Kazakhstan. In this study, hares from two regions were collected and examined to determine the types and prevalence of internal parasites. Modern genetic methods were used to identify the parasites more accurately and to explore their relationships to similar species found in other regions. Several species of helminths and protozoan parasites were detected, some of which are known to cause disease. The rates of infection varied among different parasites but were generally low to moderate. This is the first detailed study of hare parasites in Kazakhstan that combines traditional and genetic approaches. The results help improve our knowledge about wildlife health in the region and will assist in managing hare populations and protecting other animals. Understanding these parasite infections also supports efforts to maintain biodiversity and reduce risks to domestic animals and people.

## 1. Introduction

The European hare (*Lepus europaeus*) and the mountain hare (*Lepus timidus*) are well-known lagomorph species widely distributed across Europe, inhabiting a broad range of environments from agricultural landscapes to mountainous regions [1]. The mountain hare primarily inhabits forested and shrubby areas, adjacent steppe zones, the shores of reed-covered lakes and swamps, and, in exceptional cases, entirely treeless landscapes. The proportion of individuals found in these primary biotopes ranges from 51% to 63% of the total mountain hare population, depending on the year. The European hare predominantly occupies steppe, meadow, and agricultural lands as its main biotopes, although it has also been observed within forested and shrubby areas. These primary biotopes account for 80.0% to 84% of the total winter–spring population of the European hare [2].

In recent decades, significant population declines have been reported in many areas, attributed to various environmental pressures including habitat degradation, predation, and infectious diseases. Among these, parasitic infections have been identified as a critical factor influencing hare health and survival [3,4,5].

Numerous studies have documented a diverse array of helminths affecting hares, particularly gastrointestinal parasites such as *Trichostrongylus retortaeformis*, *Graphidium strigosum*, *Eimeria* spp., as well as cestodes including *Mosgovoyia pectinata* and *Taenia pisiformis* [6,7,8]. These infections can lead to severe health issues, reducing host fitness and potentially destabilizing local populations [9,10].

Beyond their direct impact on hare health and mortality, some parasites and pathogens associated with hares are of zoonotic concern, posing potential risks to human health [11,12,13]. Advances in molecular identification techniques have substantially enhanced our understanding of parasite diversity, transmission dynamics, and epidemiology, emphasizing the importance of detailed, species-specific studies [14].

Research conducted in various European countries including Italy, Germany, and Scotland has revealed considerable variation in parasite prevalence, species composition, and infection intensity. These differences underscore the role of geographic and ecological factors in shaping helminth communities [15,16,17]. For instance, high prevalence rates of gastrointestinal nematodes such as *T. retortaeformis* and *G. strigosum* are frequently reported, with significant associations noted between parasite burden and the health status of hares [18,19].

In European hares, the main parasitic pathogens identified include *Trichuris leporis*, *Passalurus ambiguus*, *T. retortaeformis*, *M. pectinata*, *Strongyloides papillosus*, *Protostrongylus pulmonalis*, *Andrya* spp., *Dicrocoelium dendriticum*, *Cysticercus* spp., *Cittotenia* spp., *Nematodirus aspinosus*, and *Eimeria* spp. [3,4,5,6,7,17,20,21]. In mountain hares, the pathogens detected include *M. pectinata*, *P. ambiguus*, *G. strigosum*, *T. retortaeformis*, *P. pulmonalis*, *D. dendriticum*, *N. aspinosus*, and *Eimeria* spp. [8,15,20,22,23,24].

Given the ecological and economic importance of hares, particularly in the context of game management and biodiversity conservation, continued helminthological research is essential. The present study aims to contribute to this growing body of knowledge by investigating the diversity, prevalence, and health impacts of parasitic infections in *Lepus europaeus* and *Lepus timidus* populations in Kazakhstan. The findings are intended to support the development of more effective wildlife health monitoring and management strategies in the region.

## 2. Materials and Methods

### 2.1. Sample Collection and Analysis

Sample collection was carried out between November 2022 and February 2025. During this period, a total of 107 hares *Lepus europaeus* (n = 46) and *Lepus timidus* (n = 61) were obtained from the Akmola and Karaganda regions of Kazakhstan.

Akmola Region is situated in the temperate zone, within the West Siberian climatic region. It features a sharply continental, arid climate with hot summers and cold winters, and significant daily and annual temperature fluctuations. Annual temperatures typically range from −19 °C to +26 °C, rarely falling below −29 °C or exceeding +32 °C. The landscape consists of a dissected small-hill terrain with predominant chernozem soils and includes forest-steppe and steppe zones.

Karaganda Region is located within the elevated Kazakh Uplands (Saryarka) and is characterized by a sharply continental, dry climate with long, hot summers and cold, snowy winters. Annual temperatures range from −20 °C to +26 °C. The region features a diverse relief including hills, valleys, dry riverbeds, endorheic, and lake basins. Its territory encompasses steppes, deserts, and mountainous areas, with mountain spurs present in the eastern part.

All animals were legally hunted by private amateur hunters in accordance with established national regulations and under officially issued hunting quotas. The species of hares belonging to the genus *Lepus* were identified based on morphological characteristics in accordance with identification guides [25].

### 2.2. Parasitological Methods

All samples were delivered to the Parasitology Laboratory of the Faculty of Veterinary Medicine, S. Seifullin Kazakh Agrotechnical Research University for helminthological dissection. The intestinal organs and muscle tissues of each animal were examined for the presence of helminths according to the method described by Skrjabin [26]. For the parasitological analysis, each animal was first examined for endoparasites with a special focus on the gastrointestinal tract. Therefore, each organ was examined externally and sectioned to search for intraparenchymatous and intraluminal parasites. Larvae of *Cysticercus* spp., adult worms of *M. pectinata*, and pinworms were isolated, washed in physiological saline, identified based on morphological characteristics [8,27,28], and preserved in 70% ethanol for further analysis.

### 2.3. Coprological Investigations

Fecal samples from each hare were collected directly from the rectum and large intestine and subjected to qualitative copromicroscopic analysis using two flotation solutions of low (50% ZnCl_2_, specific gravity 1300) and high-density Schizer’s solution with a solution density of 1.2–1.25 (454 g sugar per 1 L of water) [29,30].

### 2.4. DNA Extraction Method

Genomic DNA was extracted by homogenizing adult parasites or cysticercus in an Eppendorf centrifuge tube (Aptaca, Canelli, AT, Italy), using the standard phenol–chloroform method with proteinase K, followed by ethanol precipitation [31]. The concentration and purity of the extracted DNA were assessed by measuring absorbance at 260 and 280 nm using a NanoDrop 2000 spectrophotometer (Thermo Scientific, Waltham, MA, USA). The DNA was dissolved in double-distilled water (ddH_2_O) and stored at −20 °C until further use.

### 2.5. PCR and Sequencing

Polymerase chain reaction (PCR) was employed to assess the genetic diversity of different parasite species. Specific primers (Table 1) were used for parasite species identification and gene fragment amplification. Primers JB11F/JB12R were used to amplify a fragment of the NADH dehydrogenase subunit 1 (*nad1*) gene from *T. pisiformis* and *T. serialis*; primers COX-F/COX-R were used to amplify a fragment of the mitochondrial cytochrome c oxidase subunit 1 (*cox1*) gene from *M. pectinata*; and primers C1F/D2R were used to amplify a fragment of the *28S* ribosomal RNA gene from *P. ambiguus*. PCR reactions were performed in a 25 μL mixture containing 2× DreamTaq PCR Master Mix (Thermo Fisher Scientific, Carlsbad, CA, USA), nuclease-free water, 10 pmol of each primer, and 20 ng of template genomic DNA. Specific PCR conditions for amplifying marker regions are detailed in Table 1. Amplified products were separated by electrophoresis on a 1.5% agarose gel prepared with 1× TBE buffer, stained with ethidium bromide (8 ng/μL), and visualized under UV light.

All PCR-positive products were purified using the Quick PCR Purification Kit (QIAGEN, Germantown, MD, USA) according to the manufacturer’s instructions, then subjected to sequencing and genotyping. Sequencing was performed on a 3730xl DNA Analyzer 96-Capillary Array (Thermo Fisher Scientific, Applied Biosystems, Foster City, CA, USA). The genetic sequences obtained in this study have been deposited in GenBank under the following accession numbers: *T. pisiformis* (PV871991, PV871992, PV871993), *T. serialis* (PV871989, PV871990), *M. pectinata* (PV855722, PV855723), *P. ambiguus* (PV855725, PV855726, PV855727). The resulting nucleotide sequences were manually edited and compared with reference sequences from the GenBank database using the BLAST algorithm (https://www.ncbi.nlm.nih.gov/ (accessed on 9 May 2025)).

### 2.6. Phylogenetic Analysis

The obtained sequences were manually edited, and sequence similarity searches were performed using the BLAST algorithm (https://blast.ncbi.nlm.nih.gov (accessed on 9 May 2025)) to compare them with reference sequences from GenBank. Nucleotide sequences of various partial genes were aligned using the MUSCLE multiple sequence alignment program. A maximum-likelihood phylogenetic tree was constructed based on the Tamura–Nei model using MEGA version 11 software [32].

### 2.7. Statistical Analysis

To assess the risk of infection with parasites of mountain (*Lepus timidus*) and European (*Lepus europaeus*) hares, the odds of further infection (chances) were calculated separately for each helminth species. For all parasites in Table 2, the Fisher metric test was used, since small frequencies (<5) were observed. Values *p* > 0.05 indicate the absence of statistically significant infection signals between calendar hares. The frequency of detected parasites was statistically compared using the chi-square test, with a 95% confidence interval. The *p*-value was calculated, with statistical significance established at *p* < 0.05.

## 3. Results

### 3.1. Parasitological Research

**Figure 1:**
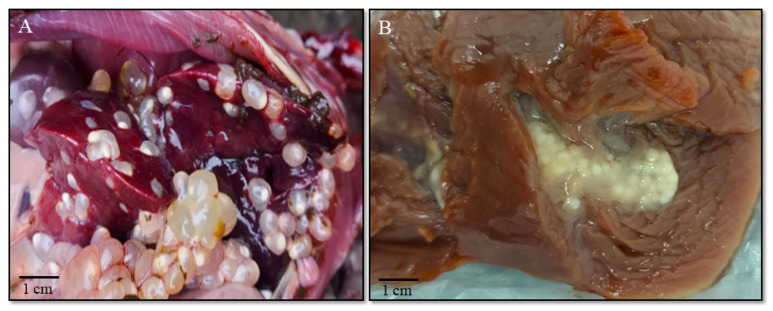
Macroscopic appearance of larval cestodes in hares. (**A**) *Cysticercus pisiformis* in the abdominal cavity of a European hare. (**B**) *Coenurus serialis* in the muscle tissue of a mountain hare.

During the study, 107 hares (*L. europaeus* and *L. timidus*) were examined. Initial inspection of the abdominal cavity and muscle tissue enabled the identification of the causative agents of cysticercosis. In 2 out of 46 European hares, similar clinical manifestations in the form of grape-like conglomerates characteristic of *Cysticercus pisiformis* (the larval stage of *T. pisiformis*) were detected in the abdominal organs (Figure 1A). The prevalence of *C. pisiformis* in European hares was 4.3% (Table 2). In 2 out of 61 mountain hares, grape-like conglomerates were also found in the abdominal organs. These structures were morphologically similar to those observed in European hares and are characteristic of *C. pisiformis* (Figure 1A). In another two mountain hares, translucent coenuri containing clusters of protoscoleces characteristic of *Coenurus serialis* (the larval stage of *T. serialis*) were detected in the muscle tissue (Figure 1B) [27]. The prevalence of *C. pisiformis* and *C. serialis* in mountain hares was 3.3% each (Table 2).

**Figure 2:**
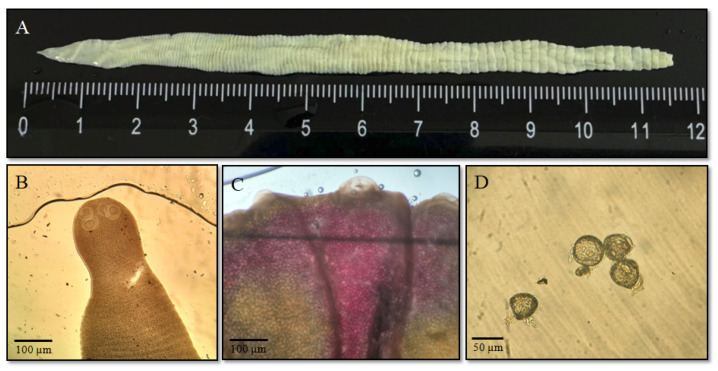
Main morphological features of the cestode *Mosgovoyia pectinata*: (**A**) general body structure; (**B**) anterior end (scolex) with suckers; (**C**) proglottids with visible genital openings and eggs; (**D**) cestode eggs.

Examination of the intestinal contents of the hare revealed two types of intestinal parasites: cestodes and small nematodes, specifically pinworms. Macro- and microscopic analyses of the recovered cestodes confirmed morphological characteristics typical of *M. pectinata*. The adult form of the cestode was characterized by a segmented body with an average length of 14.5 ± 4.3 cm (n = 23) (Figure 2A), with an anterior end bearing four suckers used for attachment to the host’s intestinal wall (Figure 2B).

The body consists of numerous segments (proglottids), which gradually increase in size toward the posterior end. Genital openings are located in the middle third of the lateral margin of each segment. Mature proglottids contain eggs (Figure 2C). The eggs are spherical or oval in shape, measuring approximately 30–40 µm in diameter, and possess a thick shell that protects them from external environmental factors (Figure 2D) [8,18,33,34]. The prevalence of the cestode *M. pectinata* was 11.5% in mountain hares and 6.5% in European hares (Table 2).

**Figure 3:**
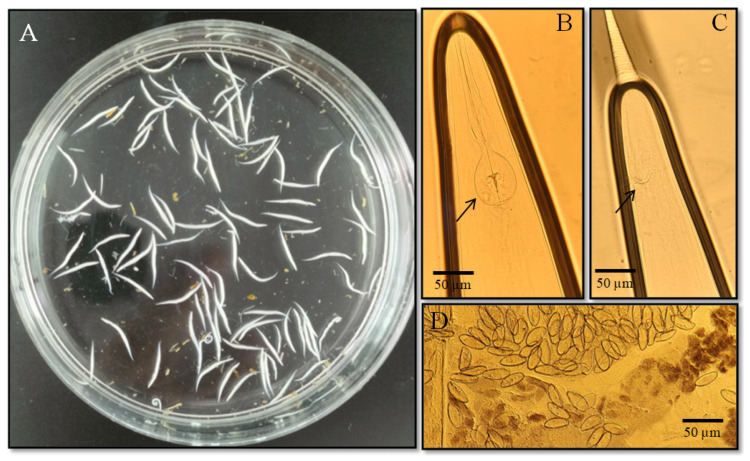
Morphological features of the pinworm *Passalurus ambiguus* found in the large intestine of hares: (**A**) general appearance of an pinworms; (**B**) anterior end showing the esophagus, bulb, and papillae; (**C**) posterior end with spicule; (**D**) characteristic oval-shaped egg with a distinct shell.

Nematodes, represented by pinworms of the species *Passalurus ambiguus*, were primarily found parasitizing the large intestine of hares. Adult females measured 7–9 mm (n = 95) in length, while males ranged from 4–6 mm (n = 71). The body was thin and whitish in appearance (Figure 3A). The anterior portion of the worm bore an esophagus, bulb, and papillae, used for attachment to the intestinal mucosa (Figure 3B). The posterior part is narrowed, and males possess a sexual spicule (Figure 3C). The eggs were oval-shaped, measuring 30–40 µm, and had a well-defined shell (Figure 3D) [18,28]. The prevalence of the nematode *P. ambiguus* was 4.9% in mountain hares and 6.5% in European hares (Table 2).

The highest odds value was observed for the mountain hare when infected with *M*. *pectinata* and amounted to 0.1296, which indicates a relatively high probability of infection compared to other helminths in this species. The similar value for the European hare was 0.0698, which indicates a lower probability of infection with *M. pectinata* in this species. Infections with *T*. *pisiformis* and *P*. *ambiguus* were detected in both hare species, with similar odds of infection in the mountain hare (odds = 0.0339) and slightly higher values for the European hare (0.0455 and 0.0750, respectively), which may indicate similar levels of susceptibility to these helminths. For *T*. *serialis*, the odds of infection were 0.0339 in mountain hares, while this helminth was not detected at all in European hares.

**Figure 4:**
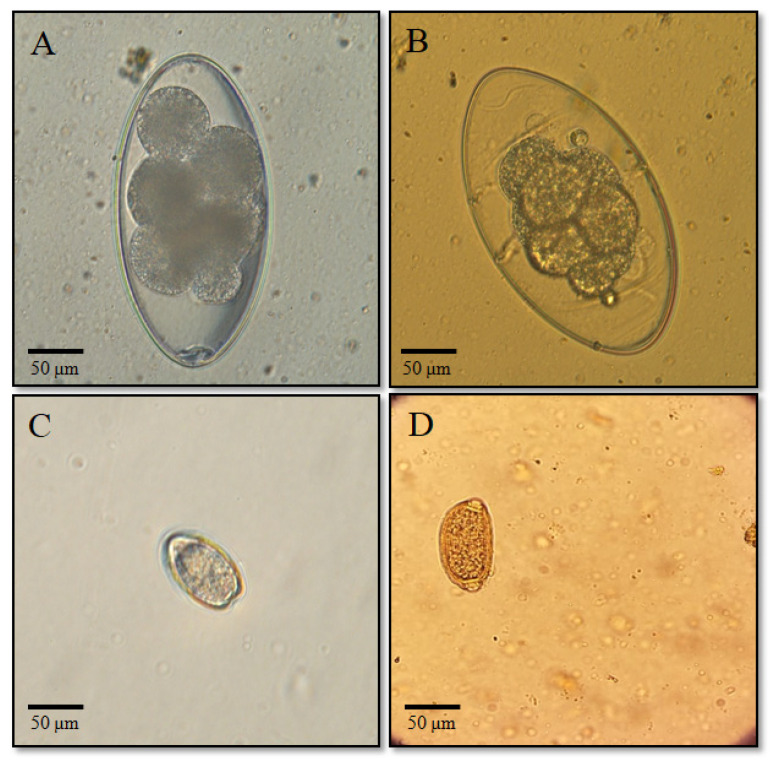
Eggs of helminths and coccidia detected in the survey: (**A**,**B**) *Nematodirus leporis*; (**C**) *Eimeria* spp.; (**D**) *Trichuris leporis*.

A study was also conducted on the prevalence of fecal-transmitted parasitic infections in hares, identified by the presence of helminth eggs and oocysts in fecal samples. A total of 63 hare fecal samples were examined. The results confirmed the presence of four pathogens: *Nematodirus leporis*, *Trichuris leporis*, and *Eimeria* spp. The results of the copromicroscopic analyses are presented in Table 3, and images of helminth eggs and coccidian oocysts are shown in Figure 4.

*Nematodirus leporis* infection is approximately 2.7 times more common in European hares than in mountain hares (Table 3). The wide confidence interval and high *p*-value mean that there is no statistically significant difference. This is due to the extremely small number of infected individuals in both groups. *Trichuris leporis* was excluded from the calculation due to the absence of infection cases in one of the hare species, which makes the statistical analysis of OR incorrect and mathematically unstable with such small samples. Although the OR values indicate an increased risk of infection in European hares, all *p*-values > 0.05 and the confidence intervals include 1. This means that the differences are not statistically significant and can be explained by random variation, especially given the small sample size, especially for *Lepus europaeus* (only 11 individuals).

### 3.2. Molecular Genetics Identification

Molecular genetics and phylogenetic analyses were conducted using partial sequences of the mitochondrial *cox1* and *nad1* genes, as well as a partial 28S rDNA region, to assess the genetic diversity and evolutionary relationships of the isolated parasites.

**Figure 5:**
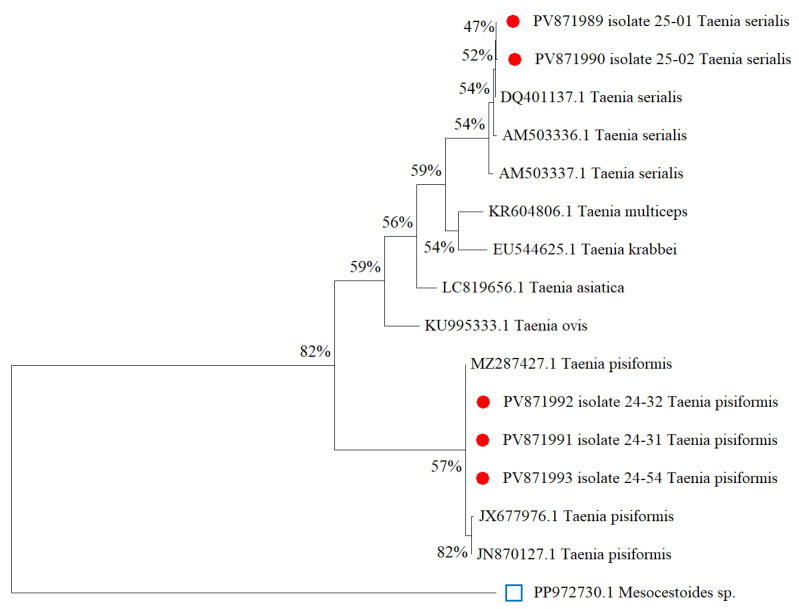
Phylogenetic tree constructed using the Maximum Likelihood method based on the Tamura–Nei model for cysticercosis species. (Red circles indicate isolates from this study).

Molecular identification of *Taenia* spp. was carried out using primers specific to partial regions of the mitochondrial nad1 gene, producing an amplicon of approximately 500 bp. The resulting sequences of *T. pisiformis* and *T. serialis* were submitted to GenBank and subsequently included in the phylogenetic analysis (Figure 5). A phylogenetic tree was constructed to determine the evolutionary relationships among *Taenia* spp. involved in cysticercosis, based on the Maximum Likelihood (ML) method using the Tamura–Nei model [35]. The analysis included 16 nucleotide sequences with a total alignment length of 893 positions. Bootstrap analysis with 1000 replicates was performed to assess the robustness of the inferred clades [36]. All evolutionary analyses were conducted in MEGA11 [32]. The *Mesocestoides* sp. (PP972730) was used as an outgroup for rooting the tree.

**Figure 6:**
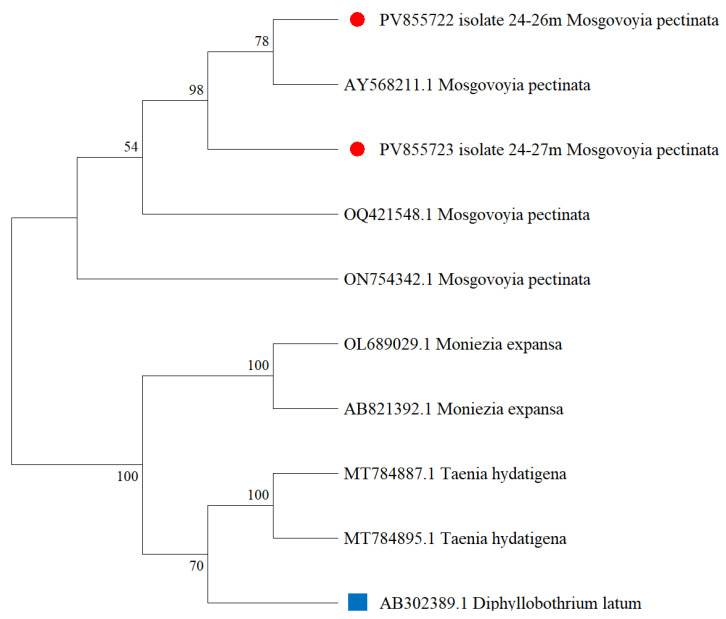
Phylogenetic tree constructed using the Maximum Likelihood method based on the Tamura–Nei model for *Mosgovoyia pectinata* species. (Red circles indicate isolates from this study).

To genetically characterize *M. pectinata*, partial fragments of the mitochondrial *cox1* gene were amplified using specific primers, generating a PCR product of around 650 bp. The generated sequences were deposited in GenBank and used for phylogenetic reconstruction (Figure 6). A phylogenetic tree was constructed using the Maximum Likelihood method and based on the Tamura–Nei model to evaluate the evolutionary relationships of *M. pectinata* with related cestodes. The analysis involved nine nucleotide sequences and a total alignment length of 13,490 positions. Phylogenetic reliability was assessed with 1000 bootstrap replicates. The *Diphyllobothrium latum* (AB302389) was used as an outgroup for rooting the tree.

**Figure 7:**
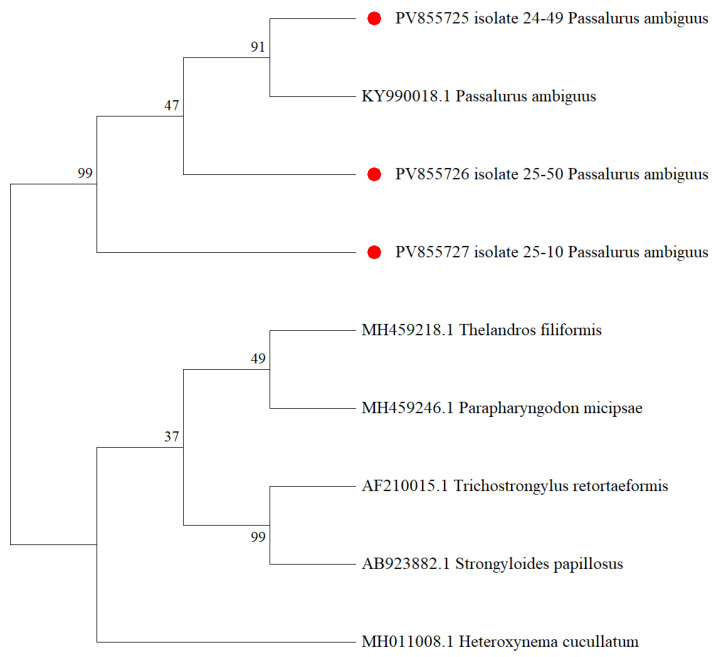
Phylogenetic tree constructed using the Maximum Likelihood method based on the Tamura–Nei model for *Passalurus ambiguus* species. (Red circles indicate isolates from this study).

The identification of *Passalurus* spp. was based on amplification of partial 28S rDNA gene regions, resulting in a ~781 bp fragment. The sequence obtained for *P. ambiguus* was submitted to GenBank and incorporated into the phylogenetic analysis (Figure 7).

## 4. Discussion

The mountain hare (*L. timidus*) and European hare (*L. europaeus*) are the most widespread hare species in Kazakhstan and are considered valuable game animals, commonly targeted in recreational and sport hunting. Their population sizes are not regulated and can fluctuate significantly from year to year, depending on various factors, including disease outbreaks. To date, no comprehensive studies on the parasite fauna of hares in Northern and Central Kazakhstan have been conducted; existing data are limited to brief reports based primarily on coprological analyses.

This study represents the first in-depth investigation of endoparasites in these two-hare species in Kazakhstan. A total of seven parasite genera were identified, including *M. pectinata*, *T. serialis*, *T. pisiformis*, *P. ambiguus*, *N. leporis*, *Eimeria* spp., and *T. leporis*. The research was geographically limited to two regions, and the sample size was not proportional to the total hare population in each area. Specimens, including hare carcasses, infected organs, and fecal samples, were obtained from private recreational hunters who voluntarily contributed to the study.

***Mosgovoyia pectinata***. The cestode *M. pectinata* (Spasskii, 1951) is a common parasite of hares and belongs to the family Anoplocephalidae, order Cyclophyllidea [37]. Its life cycle involves two hosts: oribatid mites as intermediate hosts and hares as definitive hosts [38,39]. According to various sources, species such as *Trichocalumna curva*, *Scheloribates* spp. [40], and *Ceratoppia bipilis* [41] may serve as intermediate hosts for *M. pectinata*. However, the complete life cycle of *M. pectinata*, the specific identity of its intermediate hosts, and the overall species diversity of oribatid mites in Kazakhstan remain poorly studied and represent important directions for future research. In our study, the overall prevalence of *M. pectinata* among the two examined hare species was 6.5% in European hares (*L. europaeus*) and 11.5% in mountain hares (*L. timidus*). The pathogen was detected in both the Akmola and Karaganda regions, with no significant correlation between location and infection rate, indicating a widespread distribution of *M. pectinata* across the northern and central parts of Kazakhstan. The two isolates obtained in this study (PV855722 and PV855723), marked with red circles, clustered together with high bootstrap support (89–95%) alongside reference sequences of *M. pectinata*, forming a distinct and well-supported monophyletic clade and share a similar genetic sequence with those found in Finland (AY568211) and Denmark (OQ421548, ON754342). This confirms the accurate molecular identification of the isolates as *M. pectinata*. The *M. pectinata* clade was clearly separated from other cestode taxa, including *Moniezia expansa* and *T. hydatigena*, which formed their own distinct branches with strong bootstrap support (100%), demonstrating phylogenetic divergence at the genus and species levels. These results provide strong molecular evidence for the classification of the newly sequenced isolates within the *M. pectinata* lineage.

These results are comparable to data reported from Norway and Ireland, where 12% and 14% of mountain hares were infected with *M. pectinata*, respectively [39,42]. However, the observed prevalence was considerably lower than that reported in other regions. For instance, in Finland, the average infection rate was 13% in brown hares and 30% in mountain hares [20]. In Russia, the prevalence of *M. pectinata* in brown hares varied across different regions, including the European northeast, the Kirov and Astrakhan regions, and Yakutia, where it reached up to 32%, depending on the study area [43,44,45,46]. In mountain hares, infection has also been recorded in Ukraine and Crimea, with prevalence reaching up to 58% [47,48,49]. In northwestern Iran, *M. pectinata* was found in 17% of wild European hares (*L. europaeus*) [33]. Additionally, reports of *M. pectinata* infection in hares exist from Kyrgyzstan [50], Azerbaijan [51], Armenia [52], Turkmenistan [53], and several other countries. According to the phylogenetic analysis, the isolates from this study of *M. pectinata* share a similar genetic sequence with those found in Finland (AY568211) and Denmark (OQ421548, ON754342).

***Taenia*** **spp**. The conducted studies revealed the presence of an intermediate stage of cysticercosis in hares, caused by two species of cestodes: *T. serialis* and *T. pisiformis*. The prevalence of *C. pisiformis* (the larval stage of *T. pisiformis*) in European hares and mountain hares was 4.3% and 3.3%, respectively. This was recorded in two regions of Kazakhstan—Akmola and Karaganda. *C. serialis* (the larval stage of *T. serialis*) was detected only in mountain hares from the Akmola region, with a prevalence of 3.3%. The three *T. pisiformis* isolates obtained in this study (PV871992, PV871993, and PV871991) clustered closely with reference sequences JX677976.1 and JN870127.1, forming a strongly supported monophyletic clade (bootstrap = 89%). This grouping confirms the species identity of the field isolates and suggests low intraspecific divergence within *T. pisiformis*. The clade also includes MZ287427.1, further supporting genetic homogeneity among global *T. pisiformis* strains. These findings align with previously published data by Jia et al. (2010) [54], which confirmed the monophyly of *T. pisiformis* based on mitochondrial markers. Similarly, two additional isolates (PV871989 and PV871990) grouped within the *T. serialis* clade, along with reference sequences AM503337.1, AM503336.1, and DQ401137.1. Although the bootstrap support for this subgroup was moderate (54%), the overall topology reflects expected species-level relationships. Other closely related *Taenia* species, such as *T. ovis*, *T. asiatica*, *T. multiceps*, and *T. krabbei*, formed distinct branches, confirming the genetic divergence within the genus.

*T. pisiformis* (Bloch, 1780) is a cestode with a global distribution. In its life cycle, canids serve as the definitive hosts (occasionally felids), while lagomorphs (hares and rabbits) and rodents serve as intermediate hosts [55]. The intermediate stage, *C. pisiformis*, has been detected in hares in various regions worldwide. The prevalence of *C. pisiformis* in hares has been reported as follows: 10.38% in Poland [6], 14.8% in Italy [56], 8% in Canada [57], 70% in Mexico [58], 22.7% in Belarus [59], 17.53% in Ukraine [60], 15.1% in Russia [61], and 13.33% in Iran [62]. No data regarding *T. pisiformis*-induced cysticercosis in hares has been found in open sources for Kazakhstan, indicating that the findings presented here are the first such report and warrant further large-scale studies across the country. The identity of the definitive host of *T. pisiformis* in Kazakhstan remains uncertain, as our previous investigations of helminths in wild carnivores did not reveal the presence of this species among the identified cestodes [63,64]. However, there is evidence that *T. pisiformis* circulates among domestic dogs throughout most regions of Kazakhstan [65,66], highlighting the need for further analysis, including molecular identification methods.

*T. serialis* is a rare and poorly studied parasite belonging to the *Taeniidae* family. The adult form parasitizes the small intestine of definitive hosts (carnivores, primarily canines), while the larval stage, *C. serialis*, develops in the subcutaneous and intermuscular connective tissues of intermediate hosts such as rabbits, hares, and other rodents [55,67]. Coenurosis caused by *T. serialis* has been reported in animals worldwide, particularly in Europe, Africa, the Middle East, Australia, and the Americas [68,69,70,71]. It has also been recorded in hares in Russia [46], Ukraine (Crimea) [49], Kyrgyzstan [50], and China [72]. However, no scientific data exist regarding the distribution of *T. serialis* in Kazakhstan. Given the current gaps in knowledge regarding the taxonomy and life cycle of *T. serialis* especially concerning its intermediate and definitive hosts [73] and the documented cases of human infection [74], the detection of this parasite in Kazakhstan is of particular importance. These findings highlight the need for comprehensive research into its occurrence and epidemiology within the region.

***Passalurus ambiguous***. *P. ambiguus* has a direct life cycle that does not involve an intermediate host. Eggs are deposited around the anus, and ingestion typically occurs during cecotrophy [75]. In this study, nematodes of the species *P. ambiguus* (pinworms) were identified in both mountain hares and brown hares across the entire study area, with a prevalence of 4.9% and 6.5%, respectively. Comparable prevalence rates have been reported in Finland (3%) [76], Germany (3%) [77], Iran (6.66%) [62], and Armenia (8.6%) [78]. The prevalence rates observed in our study were lower than those reported in Italy (ranging from 9.3% to 12.9%) [5,7], Egypt (45%) [28], and the United Kingdom (30%) [79]. *P. ambiguus* has also been documented in Ukraine (Crimea) [49], Russia [80], and Uzbekistan [81].

The tree (Figure 7) shows a well-supported monophyletic cluster comprising three isolates from this study (PV855725, PV855726, and PV855727), marked with red circles, which grouped closely with a reference sequence of *P. ambiguus* (KY990018.1), forming a monophyletic group supported by a bootstrap value of 91%. This confirms the identity of the field isolates and supports the intraspecific genetic homogeneity within the *P. ambiguus* clade. This finding is consistent with those reported by Hussein et al. (2022) and Abdel-Gaber et al. (2019) [16,82], who also observed the monophyly of *P. ambiguus* in phylogenetic trees based on ribosomal markers. The *P. ambiguus* clade was clearly separated from other Oxyurid nematodes, including *Thelandros filiformis*, *Parapharyngodon micipsae*, *Trichostrongylus retortaeformis*, *Strongyloides papillosus*, and *Heteroxynema cucullatum*, formed distinct branches, confirming the phylogenetic divergence from the Passalurus clade. Bootstrap values above 70% (e.g., 91%, 99%) indicate reliable topologies, while lower values (e.g., 37%, 47%) suggest moderate to low support for some internal nodes. The phylogenetic inference was based on 1000 bootstrap replicates.

*P. ambiguus* is considered the most common intestinal nematode in rabbits and hares. Although typically not highly pathogenic, outbreaks with high mortality have been reported in juvenile animals [8]. Young rabbits may suffer from heavy infections, characterized by perianal irritation, and large worm burdens can contribute to enteritis complex development [83]. Our findings expand the current understanding of the geographic distribution of *P. ambiguus* and its prevalence among wild hare populations in Kazakhstan. To the best of our knowledge, this is the first genetically confirmed report of the occurrence of *P. ambiguus* in central and northern regions of Kazakhstan. Therefore, systematic monitoring and control by national veterinary services are essential to prevent mass mortality in both domestic and farmed lagomorph populations.

**Fecal-transmitted parasitic infections**. An attempt was made to assess the main types of helminth infections present in the feces of hares collected within the study area. Coprological analysis allowed the identification of three genera: *Nematodirus leporis*, *Eimeria* spp., and *Trichuris leporis* (Figure 4, Table 3). In the mountain hare, *Nematodirus leporis* was the most common parasite with a prevalence of 13.4% (7/52) and odds = 0.1556. In the European hare, the same parasite was found with an even higher prevalence of 36.6% (4/11) and odds = 0.5714. In both cases, the *p*-values calculated using the binomial test with H_0_: *p* = 0 were <0.000001, indicating a statistically significant presence of this parasite in the populations of both hare species. For *Eimeria* spp., the prevalence in mountain hares was 3.8% (2/52), and in European hares it was 9.1% (1/11). However, in both cases, the *p*-value = 1.0000, indicating the absence of a statistically significant difference and inconclusive evidence for the presence of the parasite in the population. *Trichuris leporis* was detected in only one mountain hare (1/52, 1.9%) and was not recorded in European hares, which prevented statistical comparison. The identified pathogens are considered common and significant for lagomorphs, and their presence has been reported in various countries, including European nations [3,4,5,6,7,18], Iran [62], Ukraine [47,49], Russia [45,46], Azerbaijan [51], and several Central Asian countries [50,53,81]. The main limiting factor in conducting genetic identification of these parasites in the present study was the low intensity of infection observed in the hare samples. However, given the veterinary relevance of the detected species, especially *Eimeria* spp., the question of their species diversity and distribution among hare populations in Kazakhstan remains highly relevant and warrants further molecular investigation.

The mountain hare (*L. timidus*) and the European hare (*L. europaeus*) are closely related species, and it is expected that they share a similar set of helminth parasites. Although they inhabit the same general territory, they occupy different ecological niches. The mountain hare tends to prefer areas with dense, tall vegetation and the presence of woody and shrubby growth, while the European hare primarily inhabits open flatlands, often near outskirts of populated areas. Their diets also differ: twigs dominate the mountain hare’s diet, whereas grasses are the main component of the European hare’s diet.

These ecological differences may influence not only their exposure to parasites but also the structure of the food web in which they participate. For instance, the mountain hare is a key prey species for the lynx, which inhabits forested regions, while the European hare is more commonly hunted by wolves and foxes in steppe habitats. Consequently, the composition of definitive hosts (e.g., wild carnivores and stray dogs) involved in the life cycles of many helminths may vary between habitats.

A similar pattern is observed with intermediate hosts, particularly invertebrates such as insects and oribatid mites, whose abundance and distribution are influenced by climatic conditions. For example, oribatid mites inhabit soil, decaying plant material, tree bark, and the organic layers of forests—conditions more typical of habitats occupied by mountain hares. This may explain the higher prevalence of *M*. *pectinata* observed in *L. timidus* in our study. Thus, even when the two hare species are exposed to the same spectrum of potential parasites, differences in habitat, diet, and host interactions can result in variation in infection prevalence, intensity, and transmission dynamics.

## 5. Conclusions

The findings provide important baseline data on the prevalence and diversity of parasites in *L. europaeus* and *L. timidus*, as well as their geographic distribution and potential risk factors for parasitic infections in hare populations and other wildlife species. These results underscore the need for continued monitoring of parasitic infections in hares by national veterinary authorities, targeted outreach to inform recreational hunters about infection risks and transmission routes, and the development of management strategies for regulating hare populations in the affected regions.

## Figures and Tables

**Table 1:** Characteristics of primers used and PCR conditions.

No.	Target	Primer	Sequence	PCR Parameters	Reference
1	*nad1*	JB11F JB12R	5′-ACCACTAACTAATTCACTTTC-3′ 5′-AGATTCGTAAGGGGCCTAATA-3′	initial denaturation 95 °C 3 min	[3]
				35 cycles denaturation at 95 °C for 60 s annealing at 50 °C for 60 s extension at 72 °C for 60 s	
				final extension at 72 °C 5 min	
2	*cox1*	COX-F COX-R	5′-GATGTTTTCTTTACATTTATCTGGTG-3′ 5′-GCCACCACAAATCAAGTATC-3′	initial denaturation 95 °C 3 min	[8]
				35 cycles denaturation at 95 °C for 30 s annealing at 53 °C for 60 s extension at 72 °C for 60 s	
				final extension at 72 °C 7 min	
3	28S	C1F D2R	5′-ACCCGCTGAATTTAAGCAT-3′ 5′-TCCGTGTTTCAAGACGG-3′	initial denaturation 95 °C 2 min	[16,28]
				35 cycles denaturation at 95 °C for 15 s annealing at 50 °C for 20 s extension at 72 °C for 30 s	
				final extension at 72 °C 1 min	

**Table 2:** Prevalence and intensity of helminths found in the mountain and European hares.

Host	Species Identified	N Infected/N Examined	% Prevalence (95% CI)	N Helminth Found	Range of Intensity	Mean (SD) Intensity	Odds	*p*-Value
								**>0.05**
Mountain hare (*Lepus timidus*)	*Mosgovoyia pectinata*	7/61	11.5 (4.7–22.2)	55	3–15	7.9 (4.5)	0.1296	0.5100
	*Taenia serialis*	2/61	3.3 (0.4–11.3)	70	30–40	35.0 (7.1)	0.0339	0.5052
	*Taenia pisiformis*	2/61	3.3 (0.4–11.3)	148	34–114	74.0 (56.57)	0.0339	1.0000
	*Passalurus ambiguus*	3/61	4.9 (10.3–13.7)	103	1–87	34.3 (49.7)	0.0517	1.0000
European hare (*Lepus europaeus*)	*Mosgovoyia pectinata*	3/46	6.5 (1.4–18.0)	34	2–28	11.3 (13.1)	0.0698	0.5100
	*Taenia pisiformis*	2/46	4.3 (0.5–14.9)	107	46–61	53.5 (10.6)	0.0455	1.0000
	*Passalurus ambiguus*	3/46	6.5 (1.4–18.0)	141	5–95	47.0 (45.6)	0.0698	1.0000

95% CI: 95% confidence interval; SD: standard deviation.

**Table 3:** Study of helminths found in the feces samples of the mountain and European hares.

Host	Species Identified	N Infected/N Examined	% Prevalence (95% CI)	Odds Ratio (95% CI)	*p*-Value
					**>0.05**
Mountain hare (*Lepus timidus*)	*Nematodirus leporis*	7/52	13.4 (5.6–25.8)	0.1556 (6.68–25.27)	0.0000
	*Eimeria* spp.	2/52	3.8 (0.4–13.2)	0.40 (0.03–4.85)	1.0000
	*Trichuris leporis*	1/52	1.9 (0.5–10.3)	-	-
European hare (*Lepus europaeus*)	*Nematodirus leporis*	4/11	36.6 (10.1–69.2)	0.5714 (15.17–64.62)	0.0000
	*Eimeria* spp.	1/11	9.1 (0.2–41.3)	2.50 (0.21–30.29)	1.0000

## Data Availability

The data are contained within this article.

## Abbreviations

The following abbreviations are used in this manuscript:

N	number
bp	base pairs
CA	California
CI	confidence interval
cox1	cytochrome c oxidase subunit 1
DNA	deoxyribonucleic acid
F	Forward
MD	Maryland
ML	Maximum Likelihood
nad1	NADH dehydrogenase subunit 1
OR	Odds Ratio
R	Revers
SD	standard deviation
TBE	Tris-borate-EDTA buffer
USA	United States of America
UV	ultraviolet
